# Facilitating Family Carer Dementia Education: We All Need to Learn

**DOI:** 10.3390/healthcare12202096

**Published:** 2024-10-21

**Authors:** Anna Jack-Waugh, Susan Holland, Rhoda Macrae, Jane Mimnaugh, Debbie Tolson

**Affiliations:** 1 Alzheimer Scotland Centre for Policy and Practice, School of Health & Life Sciences, University of the West of Scotland, Glasgow G72 0LH, UK; rhoda.macrae@uws.ac.uk (R.M.); debbie.tolson@uws.ac.uk (D.T.); 2 NHS Ayrshire and Arran, Kilmarnock KA2 0BE, UK; susan.holland@aapct.scot.nhs.uk; 3 Mental Health, Learning Disability and Addiction Services, NHS Lanarkshire, Wishaw ML2 0DZ, UK; jane.mimnaugh@lanarkshire.scot.nhs.uk

**Keywords:** family carer, dementia, education, facilitation, knowledge, skills, well-being, questionnaires, focus groups, trauma-informed

## Abstract

Background/Objectives: The importance of family carer dementia education is highlighted in research, practice guidance, and policy. Less attention is paid to how facilitators learn and prepare for their role. This research aimed to explore and describe facilitator learning experiences within a bespoke Scottish Carers’ Academy designed around a theory-guided approach called Care Empathia. A healthcare and university partnership ensured integration with services and the fusion of dementia higher education know-how with clinical practice expertise. Methods: Nineteen facilitator questionnaires were completed from two Carer Academy hubs; thirteen participated in online focus groups. Results: The questionnaire findings highlighted the learning gained from being a facilitator, observing expert facilitators in action, and listening to the testimonies of family carers. The framework analysis of the focus group data identified six themes, including safe learning approaches, the art of facilitation, emotional work, team learning, and leadership. These findings highlight the importance of facilitator preparation and the advantages of co-delivery models that combine clinical and practice education expertise. Conclusion: Facilitators need to understand how to walk the talk of theory-guided educational models, be open to reciprocity in learning, and be prepared for the emotional work of delivering trauma-informed practical dementia care education to family carers.

## 1. Introduction

### 1.1. Dementia Family Caring

Within the United Kingdom (UK), almost one million people are living with dementia (PLWD) [1], approximately 70% of whom reside at home. In the context of a projected growing prevalence of dementia, ever-increasing service pressures, and an increasing move towards community-focused care, family carers are likely to remain an essential pillar in the care and support of PLWD [2]. Their care has enormous value to the people they care for and the wider society. Financially, it is estimated to equate to almost GBP 14 billion of care per annum, projected to almost double by 2040 [3].

Although family caring can bring positive and rewarding experiences for both care recipient and provider, successive studies have shown that the complex and progressive nature of dementia-related family caring is physically exhausting, associated with psychological harm and a loss of well-being [4,5,6]. A key factor that amplifies the caregiver burden is the increasing reliance and ultimate dependency upon the carer for activities of living [7].

A phenomenological exploration of the lived experience of family carers of relatives with dementia revealed that families rely heavily on trial-and-error approaches to manage extremely complex symptoms and situations [8]. An observational study that examined the characteristics of family carers’ skills also found that family carers of PLWD often need to self-develop skills and coping mechanisms to manage new situations and progressive changes [9]. Among the practical challenges is knowing what to do when a relative with dementia is distressed or resists care [10]. When challenges associated with the practical aspects of care provision or caring dilemmas feel unsurmountable, family caring is difficult to sustain. Such situations fuel premature or inappropriate admission to long-term care or crisis-led hospital admissions, the risk of which is known to increase as dementia advances [11].

Although there is a plethora of information available, information alone is not sufficient to support family carers to use approaches within the complexities of dementia care at home, particularly given the known psychological harms of and impacts on family carers’ well-being. The need for access to family carer education programmes that improve knowledge and caregiving skills are well recognised within national policies and guidelines [12,13,14,15]. Despite this, few education programmes focus on providing family carers with the skills and knowledge to manage fundamental care needs and sustain family caring [10,16]. This lack means many family carers remain unprepared for the complex and progressive changes that occur during the dementia illness trajectory [17].

Mindful of the call for evidence and skills-based education for family carers, a new and innovative model of dementia family carer education, within what would become the Alzheimer Scotland Centre for Policy and Practice (ASCPP) Carers’ Academy, was co-designed with family carers, Alzheimer Scotland Dementia Consultants (ASDC’s), and clinical practitioners. This paper focuses on facilitator learning experiences from Hubs 1 and 2 of the Carers’ Academy.

### 1.2. The Carers’ Academy

The first ASCPP Carers’ Academy programme was established in 2018 at a university campus with its constituent NHS service Campus (Hub 1). Additional programme hubs were established in 2021 (Hub 2) and in 2024 (Hub 3) at different campus sites with respective local NHS services. As far as we know, it is the only university-based, skills-based education programme for family carers.

This bespoke two-day programme has been specifically designed to support the educational and practical skills-based learning needed to support and sustain family care for a relative living with dementia.

The focuses are as follows:

- Enhancing knowledge and understanding of the effects of dementia.
- Developing practical skills in the fundamentals of care and caring.
- Facilitating opportunities for peer-to-peer learning.
- Introductions to community services and support and making healthcare connections.

Content and teaching resources for Day 1 and Day 2 are standardised across the delivery Hubs, and sessions are led by core facilitators supported by sessional facilitators from local services. Sessional facilitators (such as specialist nurses, physiotherapists, occupational therapists) make specific contributions determined by their expertise and practice role. All new facilitators begin their learning and facilitation experiences on Day 1 before moving on to Day 2.

In keeping with the relational aspects of care, the ASCPP Carers’ Academy model recognises family carers as partners in dementia care. Establishing therapeutic relationships between family carer attendees and the facilitators led by the local ASDC, supported by Alzheimer Scotland staff and NHS Teams, is central to programme planning, delivery, and follow-up support. This approach includes access to support before, during, and after programme attendance with an ASDC or senior practitioner. The practitioners can make urgent referrals if required, connect people with appropriate services, and provide introductions to local community support. To our knowledge, the integration between the education programme and local services and community support is unique.

Learning is embedded in the principles and values of trauma-informed, relationship-, and person-centred care and is underpinned by UWS’s signature Care Empathia approach [18]. This approach creates the conditions to deepen the understanding of dementia and its impact on brain and bodily health, appreciate emotional aspects of care and caring to strengthen empathy, and gain skills required for practical actions and safe care solutions. Integrating theory and practice with family experiences while providing evidence-based expertise is at the programme’s core. The approach to learning is tailored to meet the individual needs of the caring situations of attendees during each programme session.

## 2. Materials and Methods

### 2.1. Study Aim

To describe and understand the experience of facilitators learning to deliver family carer education within a bespoke Scottish Carers’ Academy.

### 2.2. Participants

The voluntary participants were 19 Carer Academy facilitators from Hubs 1 and 2, who worked in the Carers’ Academy either on a core or sessional basis and delivered the programme over a period of 12 months. All facilitators from both hubs were invited to participate. They included NHS Senior Mental Health and Adult Nurses, Alzheimer Scotland staff, Senior Allied Health professionals (AHPs), and dementia education specialists from higher education (academic and practice education experts).

### 2.3. Data Gathering Methods

From 2023 to 2024, all Hub 1 and 2 facilitators were invited to complete anonymous facilitator questionnaires at the end of Day 1, which focused on Dementia Essentials. Nineteen facilitators’ questionnaires were returned.

The questionnaire focused on three areas:

1. Reflection on their expectations about their Day 1 facilitation experience.
2. Aspects of the Day1 session that they found thought-provoking in relation to their own learning.
3. The areas of their own learning for development.

To capture learning over time, two hour-long online synchronous focus groups six months apart were facilitated to explore facilitators’ individual and shared learning experiences over time. A total of 13 facilitators took part in focus group discussions. Both focus groups 1 and 2 involved nine participants, with five opting to participate in both focus groups. Ideal for expert practitioners, focus groups support the exploration of a specific phenomenon, providing an opportunity to probe deeply into experiences, understanding, and insights [19]. The focus groups were conducted virtually and recorded on Microsoft Teams. Then, the transcript was downloaded and anonymised.

The reflective questions used in the focus groups are shown in Table 1

### 2.4. Ethical Considerations

The UWS HLS School Ethics Committee granted ethical approval. Participants were recruited from the facilitator groups of Hub 1 and 2 by AJ-W, who was not involved in Carers’ Academy delivery or design and led the data collection. Participants were provided with participant information sheets, and they signed and returned consent forms before data collection commenced. All data collection, handling, and sharing approaches complied with the six principles of Article 5 of the GDPR [20] and the University’s Guidelines for Ethical Practice in Research and Scholarship [21]. The research lead was the data custodian.

### 2.5. Data Analysis

The facilitator questionnaires were subjected to thematic analysis. Patterns were sought, and themes were identified under each question. Qualitative data from focus group meetings underwent framework analysis [22]. Framework analysis stages 1-7 consisted of familiarisation with data from both focus groups, initial coding, and development of the analytical framework. Trauma-informed principles provided a priori themes for the framework development. In stage 3 (coding), however, using the broadly deductive approach recommended [22], it became apparent that coding using the TI principles did not represent the rich themes in the data. An expanded framework, which subsumed the TI principles, was then developed for the second phase. The second phase was to apply the analytical framework by charting the data into the framework matrix and agreeing on the final themes in the framework with aligning data [23,24]. Agreeing on the final themes was undertaken with a senior researcher reviewing the framework matrix containing the aligning data. Minor editing of the themes to increase understandability and congruence with the original transcripts was the final stage of analysis and the presentation of findings.

## 3. Findings

This section presents the questionnaire findings first, followed by the focus group findings. Both are accompanied by illustrative verbatim quotes.

### 3.1. Facilitator Questionnaires

Facilitators expected an interactive and relational approach to teaching and supporting carers. Some were worried about low attendance rates affecting interactivity and the learning experience. Developing relationships with other facilitators was seen as important to improve input quality and the Carers’ Academy development. Some recognised the uniqueness of the cohort of attendees and in-session dynamics.

*To provide carers with information and tools to help them along their journey. I expected it not to be the same as previous sessions, as I have learned it is different every time, depending on carers. I hoped that carers would be interactive during the day.*

Core facilitator Hub 1.1

In response to what two aspects of the day they found thought-provoking concerning their own learning, almost all reported they had learnt something from the day. Around two-thirds reported that specific aspects of the programme enhanced their learning about dementia, and they had greater insight into the experiences and challenges of family caring. Many reflected that they learnt new approaches to teaching and learning or enhanced their knowledge about how dementia can be experienced.

*To provide carers an opportunity to learn and be able to ask individualised questions relating to their loved one with dementia. Each and every time I come to the C.A. I learn something new from the carers and facilitators.*

Core facilitator Hub 1.2

Facilitators observed that carers face challenges such as juggling work and caring responsibilities, lack of information and support, and poor communication between services and professionals.

*That some carers are of similar age to myself and trying to juggle the caring role with family/work life. The struggles carers face trying to obtain information on rights and entitlements.*

Core facilitator Hub 1.4

The other main pattern that arose was how emotional the experience of facilitating and hearing carers experiences was for facilitators and carers. Some reflected on how they needed to give more thought to ensuring all voices were heard and supporting and managing the powerful emotions expressed within the group.

Facilitators aimed to develop their learning further by learning more, improving facilitation skills, and informing practice. Half of them planned to refresh or increase their knowledge on topics such as depression, medications, cognitive stimulation, hydration, rights and entitlements, and locally available services and support. AHPs focused on disciplinary-specific knowledge.

*To further my knowledge in the assessment of older people’s balance and mobility, particularly those with dementia.*

Sessional Facilitator Hub 2.2

Just under half wanted to improve their facilitation skills. This included increasing or adjusting their preparation for the day to ensure that their teaching and learning approach was accessible, inclusive, and flexible for the individual learning styles of the day’s carers.

*Return to asking carers to write down specific issues and give them time to reflect during breaks about what information they want and then tailor afternoon practical session better.*

Core Facilitator. Hub 1.5

Around a quarter of the responses related to informing practice. Some of these were about using the learning gained from being a facilitator of the Carers’ Academy and using it in their own practice. For a small number, their experience of being a facilitator and hearing about carer experiences was a ‘call to action’ to influence or lead improvements in practice.

### 3.2. Focus Groups

Thirteen Carer Academy facilitators participated in the focus groups; five participants attended both focus groups. All were recruited from the same facilitator sample who completed the facilitator questionnaires.

The focus group findings demonstrate the interplay between the family carers and the facilitator’s co-creation of learning. Six themes were identified that reflected the gradual embodiment of the underlying learning model (pedagogy) and the reciprocity within the learning experience of the facilitators themselves. The six themes were as follows:

- Thinking about learning safely
- Doing it, discussing it, and feeling it
- Developing collaborative insights
- The emotional work of facilitation
- Being responsive to caring situations
- Leading, learning, and working as a team

#### 3.2.1. Thinking About Learning Safely

There is a process for referrals, preparation, and bringing family carers to the Carer Academy. These preparatory interventions ensure that family carers and facilitators can undertake challenging learning in a safe physical and psychological environment. The focus group provided key examples of the essential nature of this support.

*So I think for me, that’s what our ethos has always been about, keeping people safe.*

*And I think we were challenged around that recently to think, how do we do this? Do we support it, is a right thing to do? If we’re going to support it, how do we keep somebody safe?*

*So I think for me that’s the kind of been one of the standout things over the last few months that a unique kind of situation that we have found ourselves in.*

FG 11 Nurse

The post-academy intervention was equally important. The senior clinician who supported referral and access to the Carer Academy provided a follow-up phone call and responded to the family carers’ needs raised during and after the day.

*That’s an opportunity that we offer and again then even that maybe the next day, it might be a call from myself or even to touch base to see how things are…… and to have that more intense communication after the day whereas normally if everybody’s OK, it would be that kind of 1 phone call a week later, how did you enjoy the day?*

FG1 Nurse

#### 3.2.2. Doing It, Discussing It, and Feeling It

The learning outcomes of the Carers’ Academy are achieved by structuring a series of interconnected but distinct learning activities. Family carers have choice and control over their level of participation and are invited to participate in the Care Empathia approach at a level they find comfortable. The facilitators reflected on how the learning activities create lightbulb moments for the family carers when unexplained behaviours make sense when the experience of people with dementia is brought to life. The starting activities, including photo elicitation, set the scene for everyone.

*And I remember one care because fairly early on and she picked the little orangutan baby monkey in a cage and she said this is exactly how I feel. And I feel without that card, she would have probably said we can find it hard, but I think that picture of that monkey in the cage, …..that enabled her to open up more than she would have if somebody had just said to how you are doing as a carer. that she felt trapped and she felt like her life had changed. She had no control over anything.*

FG5 Dementia Advisor

Drawing from the theory of Care Empathia, where learning about the complexity of dementia occurs through the head, heart, and hands, family carers are asked to trace a mouse through a maze in a mirror image. This exercise helps demonstrate the effect of dementia on both brain and bodily function and the potential impacts of poor communication.

*They are trying to concentrate, and then we put a time on it, and count down, and you can just see how they start to understand that actually how much people with dementia must have to concentrate when they’re trying to do something. How disruptive is it to their concentration when people start talking and asking them other things?*

*And then when somebody starts to rush them, how they either freeze or it just goes totally wrong. So I think it’s just, it’s such a simple activity, but so impactful and powerful.*

FG1 Nurse

Domus, meaning home, is a room established in each Carer Academy hub. Designed as a homely environment, the Domus is equipped as a dementia-enabling environment. Equipment and technology are set up for family carers to learn and evaluate whether equipment may support them, with many carers sharing their learning.

*when we go into the Domus room, I’ve noticed the difference between when we do get able to go in there and when we’ve stayed in the room……..we’ve entered the room, an awful lot of the conversation starts about technology as well. And we talk about mobile phones and GPS and often not physical things that are in the room, but it generates conversations from people …… So they’re able to share their knowledge with other people as well.*

FG4 AHP

Family carers have a supporting mobility and movement session with a dementia specialist AHP. This provides a different lens and voice for family carers to understand their family members’ experience with dementia, therefore further contextualising those previously unexplainable behaviours and symptoms.

*And so it’s really nice to be able to explain, the links between the difficulty with brain function and why that has an impact on mobility and why they’re seeing, the issues that they’re seeing with people speak, getting frozen when they’re walking or people having difficulty lifting their feet properly when they’re walking, because that can be quite frustrating for carers without having a reason for it.*

*But the links between brain damage and poor motor control are sometimes forgotten about, and among all those conversations in which is understandable because it’s not the main focus. So we get lots of light bulb moments in that little session about why that’s a difficulty.*

FG9 AHP

#### 3.2.3. Developing Collaborative Insights

Mutual family carer learning and sharing are staged through learning activities, which provide family carers with opportunities to share their experiences, empowering each other with their shared knowledge and learning. Facilitators discussed a range of cognitive and emotional responses through these processes, including when family carers shared their learning in their previous or current caring situations.

*So if it’s husband and wife, the relationship is completely different now it’s they’ve got to grieve for that romantic relationship that was once there. Or if it’s mother and son they’ve got to, they’ve lost that relationship where you would look to your mum for help or advice or whatever. So you they’re almost feeling that they’ve lost the person, but being able to regain some empathy and regain that relationship of that closeness again, I think that’s a huge thing for a lot of people. I think sometimes, if people are completely honest, they start to dislike the person that they’re looking after, I can fully understand why. And then I think that’s coming to the Carers Academy, finding out what’s behind the behaviours and the different things allows them to regain some of that love that they had for the person before.*

FG12 Nurse

The nursing contact enacts the creation of safety, choice, and control before the Carer Academy. Creating an educational space where family carers feel safe enough to discuss behaviour or emotional reactions that they do not understand or can misinterpret is the start of the learning at the Carer Academy. From that point of safety, family carers come together as a group and start to assimilate the education about the impacts of dementia on their family member, developing a deeper understanding of the actions and reactions of the people they are caring for. Seeing the ‘light bulb moments’ when understanding is achieved stimulates applying the learning to past or in-the-moment caring experiences. Through discussions between the family carers, supported by facilitators, the applications and evaluations of that knowledge are seen and heard.

#### 3.2.4. The Emotional Work of Facilitation

Being a small, prepared team was valued as facilitating trust and safety between facilitators and trust and safety between facilitators and family carers attending the day. Part of this was attending to and managing situations that created an emotional response in the family carers and facilitators that were distressing, sad, tender, hopeful, or required an immediate professional response outside of the carer academy.

*definitely, I think that Ice breaker section is a fantastic session and again they see us as humans because we be open and honest and the things that we see as well at that day at that point of the day.*

Nurse FG1

*Now, if I’m doing Carers Academy, there are no visits getting done after that because, I’m emotionally drained quite often by the end of it if I’m honest. And that’s I think that’s something we’ve always prepared folk for. That’s really key to those who are involved and aware that you need to look after your own emotions.*

FG4 AHP

In the focus groups, facilitators described responding to extreme grief, desperate loneliness, failure of services, and guilt when family carers described what they defined as mistakes with their people with dementia. These reports required facilitator focus, self-control, and compassion to ‘hold’ the psychological safety of the individual and group members. Quotations typifying these events cannot be used in the article as family carers and people with dementia could be identified due to the unique circumstances the family carers experienced.

Being able to come together as an expert team, deliver the Carers’ Academy Day, and reflect afterwards to ensure that problem-solving occurred at the moment and learning was fed forward to the next session. Managing facilitator emotions, the emotions and disclosure of family carers, and work time during and after each Care Academy was a vital strategy developed as part of the Carers’ Academy facilitation.

#### 3.2.5. Being Responsive to Caring Situations

Flexibility and choice were important for responding to family carers’ needs each day. As experts in their practice, the facilitators understood that they had an outline plan for the day and learning outcomes that must be covered; this could potentially change in line with the family carers’ responses.

*So we come up with some ideas about what carers might want to hear about things like self-directed support, adult care support plans, etc. However, quite often at the session, the discussion may go in a slightly different direction. For example, we’d prepare to speak about those two things one week, and we ended up speaking about the Herbert Protocol and Purple Alert for all of the session because that was what the carers in that group hadn’t come across those things. So I think for us it’s about the bit about being flexible.*

FG5 Dementia Advisor

The facilitators recognised the importance of achieving the learning objectives at the pace of the family carers. They knew that part of achieving the learning objectives included family carers having time and space to talk about what was important to them and doing that in a manner that enabled them to choose when to speak and when to relax and have fun.

*And I think as long as there’s definite ways you can mix up what people are doing, it keeps folk interested and it keeps them and invested in in what you’re trying to get across. So I think that’s really important. And again, it’s just another skill that these facilitators have is, is looking at the timetable and being able to mix it up a bit so that you’re getting some of the serious messages across and then just lightening the mood a little bit.*

FG9 Dementia Advisor

#### 3.2.6. Leading, Learning, and Working as a Team

Findings from the focus groups illuminated the essential nature of the mentoring and teaching skills when bringing newly formed teams together, including modelling expertise in facilitating the intertwined learning activities and engaging in reflections with the teams after each day.

*But for me, what I think is key is when [the facilitator] starts off presenting, they set out the agenda for the day. So people are then people are attending, quite nervous, a bit daunted, not knowing what the what’s ahead of the day. …….... We’re going to then break for lunch and then this is what we’re going to do. So, people who are within that group are saying this is what the agenda is. As for trying to guide the conversation, she is just an expert at that.*

*That’s just something that she can naturally do to bring it back. So, I think a big part is the key, but obviously, she’s got outstanding skills to bring it back.*

FG10 Dementia Advisor

The practice of all the facilitators reflecting together after each Carers’ Academy was felt to be essential to their developing knowledge, competence, and confidence.

*….. it’s been it was evolving, in the beginning so it was really good to get the chance to go through that with somebody else and talk about the stages and talk about the things we’re trying to discuss and obviously for someone to put their own spin on it. And really analyse what we talk about and think about it from a learning perspective as well as what we’re trying to put across the carers.*

FG3 AHP

All focus group participants reflected on how working as a team during Carers’ Academy delivery developed and extended their dementia expertise.

*I was able to learn I’ve actually learned an awful lot about dementia because I’ve come from a general nursing background. So, although I’ve nursed people with dementia, obviously, over the years, it’s not something that I’ve done a large amount of increased training. With that I’ve learned a huge amount about dementia from going to the Carers Academy in itself.*

FG12 Nurse

The Carers’ Academy was crafted to meet the needs of all potential family carer participants. However, the expertise within the room could flexibly tailor the education to the individual experiences and needs that their family carers brought to the programme on the day. It is through learning from expert modelling that they have trauma-informed educational approaches, systems, and experiences to be able to respond to the carers in the moment and foster their learning and the learning of all in the room.

*So, I think the program has been crafted over time to on that day one resonates with everybody but allow the professionals within that expertise to answer the particular problems that some family members may be experiencing.*

FG 11 Nurse

Developing and evolving as a team was an ongoing process discussed and agreed on by the focus group participants. This evolution resulted in positive outcomes for the family carers and facilitators attending the day.

*I think I want to acknowledge how thankful I am and how we feel about the physical health section, becoming a kind of core part of the Carers Academy, and being recognised within the core teaching team. But I think I think that was my reflection it’s just that it’s felt like we’ve been talking for a very long time about physical health in dementia, and it feels like having this as a core thing. It really validating for that and really key to supporting Carers to understand that.*

FG3 AHP

The commitment to quality family carer education both challenged and extended the knowledge, skills, and emotional capacity of these health and social care professionals who came to the Carers’ Academy as experts. The ongoing collaborative development of the model of the Carers’ Academy held the family carers’ needs centrally, requiring clear clinical and educational leadership to make ‘live’ the trauma-informed principles within structured educational activities. Additionally, a wish to continually reflect, develop, extend, and refine the facilitators’ professional practice in supporting and learning from family carers is infused throughout the findings of this study.

## 4. Discussion

There are a plethora of studies reporting the benefits of family carer psychoeducation interventions [25,26]; less numerous are studies focusing on dementia care knowledge and practical skills [10,16]. Interestingly, although many of these studies note the pedigree of trained facilitators, often stating graduate status, their role is project-specific and time-limited, focussed on testing a protocolised intervention. Not surprisingly, attention to the details of facilitator training in these reports is scant. In contrast, our work prepares facilitators to deliver family carer dementia education using a theory-guided model within an integrated service-education Carers’ Academy. The intention is to prepare dementia practitioners and experienced dementia educators to form stable and sustainable local delivery teams.

While evidence calling for family carer education is compelling [14], how this should be provided and by whom is unclear. In recent years, the complexity of dementia care has been highlighted, giving impetus to frameworks such as the Scottish framework, Promoting for Excellence, which charts the incremental levels of knowledge and skills required of the health and social care workforce, incrementally rising through skill levels up to that of the expert practitioner [27]. If we view family carers as partners in care [28], they deserve the complexity of family-based dementia caring to be recognised, and they deserve access to appropriate dementia education delivered by knowledgeable and capable facilitators.

Only four studies in a systematic review of 152 dementia workforce education studies reported outcomes associated with positive engagement with family carers [29]. Surprisingly, it was unclear how these development programmes equipped practitioners for their potential role in delivering family carer dementia education. Our work has highlighted the importance of facilitator preparation and recognising the emotional work that accompanies facilitating family carer dementia education. Trauma-informed approaches and the inevitable disclosure of caring difficulties during dementia family carer education make facilitator support through reflective practice opportunities important considerations.

It is well documented that expectations of being a caring person pervade the complex experiences associated with family caring. Furthermore, there is a tendency among family carers to put the perceived needs and emotions of the person with dementia above their own. Using the lens of emotional work, this complexity of experiences associated with family carers’ emotional expectations and their quest ‘to do the right thing’ was explored [30]. Even for practitioners with a knowledge of the complex brain and bodily changes arising from dementia progression, *getting it right* in the moment can be challenging. New Carers’ Academy facilitators were advised not to feel they should or could provide all solutions. Rather, they should see their facilitation role as helping participants gain insight and empathy into dementia-related changes, including behaviour changes, and use this insight and what they know about the person to explore and consider person-centred approaches.

The theme *Being Responsive to Caring Situations* necessitates an invitation to the attendees to share with the group of peers something about their own situations, challenges, and rewards. Navigating the inevitable emotionality of this requires interpersonal skills and an ability to adapt and respond in the moment with an understanding of the need for flexibility in planned learning outcomes. While some family carer accounts enrich opportunities to *Develop collaborative insights*, the facilitator has to balance the interests of the individual who is sharing and be responsive to the impact on the group. This is where facilitators with differing levels of confidence, competence, and knowledge can, working as a team, gently steer using a conversational style to validate someone’s experience and thoughtfully move forward to illustrate a useful learning point for others. Such acknowledgement, validation, and normalisation of carers’ experiences are widely reported in the group psychoeducational literature [25,26]. Furthermore, vicarious learning between peers can give confidence in finding solutions that have worked for others while simultaneously supporting and learning from each other [31]. Importantly, as our facilitators came to appreciate through *Thinking about learning safely*, they had a collective responsibility to use their clinical judgment to elaborate or qualify something shared to promote safe and appropriate solution finding aligned with their growing familiarity with individual caring situations. The diversity of expertise among the facilitators and teamwork ensured a trauma-informed educational approach when responding to carers in the moment.

The findings from the questionnaires and focus groups in our research suggest parallels between the *emotional work* of facilitators and the family carers who are there to learn. Undoubtedly, the facilitators put the perceived needs and emotions of the attendees above their own. The focus group participants frequently described feeling emotionally drained at the end of the session, with the need to come together as a team immediately afterwards to reflect, share feelings and observations, and feed any learning through to the next session. There was also a sense of awe in how the expert facilitators navigated the learning experience, weaving in in-the-moment responses to family carers’ reactions and comments, oscillating between individual and group, stewarding the peer-to-peer learning, and refocusing to keep session momentum and focus. Interestingly, from questionnaire responses, most underestimated the intensity of the emotional experience involved in facilitation and the complexity of the group dynamics within learning. As experts in their field, they were challenged in their emotional capacity and level of knowledge and skills.

Several participants acknowledged the importance of deepening their understanding of dementia and dementia care, echoing a sense of ‘not being an expert’. This sense of not knowing was strong for some, yet their managers identified the facilitators because of their practice competence and dementia expertise. This loss of confidence was not, however, unexpected given that they were, for the first time, putting into practice a sophisticated learning model based on Care Empathia. For most of the new facilitators, this approach was novel. Discomfort in learning is a well-known phenomenon, particularly among experienced professionals, but it is an essential step to understanding that there may be a different way of doing things that are superior to their current practice [32]. For participants in this study, the discomfort they experienced probably had more to do with their lack of familiarity with the underlying family carer educational model (pedagogy) than with the absence of dementia care expertise.

A key lesson from our work is that delivering a transformational dementia family carer education requires a theory-guided approach and an explicit practice-based learning pedagogy. A partnership approach to delivery that brings together expert dementia practitioners with expert practice-based educators strengthens family carer dementia education. Importantly, attention should be afforded to equip all facilitators with the confidence to deliver family carer education aligned with the underlying theory and pedagogy. The emotion work inherent within dementia care and the impact of the psychological distress, including anticipatory grief that accompanies dementia family caring, pervades the learning experience. Accordingly, facilitators need support themselves and opportunities for reflective practice and support for their emotion work.

### Merits and Limitations

The qualitative approaches used in this work complemented one another, with the relatively brief responses typical of questionnaire data being illuminated through the fuller focus group discussions.

The Carers’ Academy approach to family carer education is distinctive and rests upon a signature learning model. Caution is advisable as some of the insights into the experience of facilitator learning may not be generalisable. However, this is offset by being theory-guided (person-centred, fundamentals of care, trauma-informed) in ways aligned to contemporary best practices in dementia care.

## 5. Conclusions

Facilitating dementia family carer education requires clinical practice expertise and confidence in delivering dementia practice-based education. Furthermore, a theory-guided approach underpinned by an appropriate transformational learning pedagogy directly informs and shapes the approach to facilitation. Hence, family carer dementia education co-delivered by practitioners and educators combines their respective dementia expertise and has many advantages. Facilitation practice is enhanced through training that promotes the development of shared practices and embraces learning with and from family carers. Peer-to-peer learning occurs not only between the family carers but also between staff facilitators. Being receptive to reciprocity in the learning experience augments staff facilitators’ opportunity to learn directly from those living the experience of family caring, deepening understanding and empathy. A recognition of the emotional work of facilitators and strategies, such as reflective practice sessions, should be considered and evaluated for effectiveness by providers of dementia family carer education. Further research is required into the impact of expert-facilitated education programmes on the well-being of family carers and the people with dementia that they care for. We all have much to learn about and from dementia family carer education.

## Figures and Tables

**Table 1:** Focus Group Questions.

From your experiences at the Carers Academy over the last four months,
Tell me about the delivery of the sessions on the day and what went well? Can you explore where the delivery team was particularly successful? Can you give examples of positive responses from the family carers who attended? Can you discuss what key issues you heard from family carers that you are able to share? What are your personal or team learning moments?

## Data Availability

The original contributions presented in this study are included in the article; further inquiries can be directed to the corresponding author.
